# A self-training spiking superconducting neuromorphic architecture

**DOI:** 10.1038/s44335-025-00021-9

**Published:** 2025-03-04

**Authors:** M. L. Schneider, E. M. Jué, M. R. Pufall, K. Segall, C. W. Anderson

**Affiliations:** 1 Applied Physics Division, National Institute of Standards and Technology, Boulder, CO 80305 USA; 2 Department of Physics, University of Colorado Boulder, Boulder, CO 80305 USA; 3 Associate of the National Institute of Standards and Technology, Boulder, CO 80305 USA; 4 Department of Physics and Astronomy, Colgate University, Hamilton, NY 13346 USA; 5 Department of Computer Science, Colorado State University, Fort Collins, CO 80523 USA

**Keywords:** Superconducting devices, Applied physics

## Abstract

Neuromorphic computing takes biological inspiration to the device level aiming to improve computational efficiency and capabilities. One of the major issues that arises is the training of neuromorphic hardware systems. Typically training algorithms require global information and are thus inefficient to implement directly in hardware. In this paper we describe a set of reinforcement learning based, local weight update rules and their implementation in superconducting hardware. Using SPICE circuit simulations, we implement a small-scale neural network with a learning time of order one nanosecond per update. This network can be trained to learn new functions simply by changing the target output for a given set of inputs, without the need for any external adjustments to the network. Further, this architecture does not require programing explicit weight values in the network, alleviating a critical challenge with analog hardware implementations of neural networks.

## Introduction

Neuromorphic computing hardware draws inspiration from the way the human brain processes information to improve computational efficiency and broaden the potential methods of computation beyond typical digital logic^1–4^. There are many reasons to look to the brain for computational inspiration. Using roughly 20 Watts of power, the human brain is an extremely energy efficient computational system. In addition, with its highly parallel architecture, the brain is both fault tolerant and can perform certain tasks with surprising speed given the typical neuron firing rate of a few hundred hertz. Perhaps the most intriguing properties of the brain are its adaptability and capability for learning new tasks with minimal supervision and incomplete training data. With these capabilities in mind, we have developed an architecture that is self-training, generalizable, fast, and energy efficient.

Superconducting hardware lends itself to a neuromorphic approach in part because of the natural spiking behavior of the Josephson junction (JJ), similar to a neuron, and the near lossless propagation of these spikes on superconducting transmission lines, similar to axons^5^. Because of potential improvements in energy efficiency and speed, a superconducting digital logic family has been developed using these JJ based spikes, called single flux quantum (SFQ) logic^6^. These superconducting circuits can operate at speeds in excess of 100 GHz and are extremely energy efficient^7–9^. For example, the typical spiking energies of the JJs modeled here are less than one attojoule. Once a cryogenic system is scaled beyond the initial research and development phase, it takes less than 1000 W of wall power to cool 1 W of power dissipated at 4 K. This results in SFQ spiking energies that are still more than an order of magnitude lower than the human brain’s 10 femtojoules per spike, even when accounting for the energy overhead required to operate at 4 K.

In part because of the characteristics noted above, there has been a recent interest in superconducting neuromorphic computing. Several different implementations of superconducting neuron or soma cells have been simulated^10–12^ and implemented in hardware^13–15^. JJ neurons that take advantage of the high-speed dynamical interactions for greater biological realism have also been implemented^16–18^. Various superconducting synaptic cells have been proposed including those based on hybrid superconducting spintronics^19^, biased superconducting quantum interference devices (SQUIDs)^13,20^, and stochastic JJ synapses where the weight is transformed into a probability^21–23^. Using these building blocks small JJ based circuits have been shown to exhibit spike timing dependent plasticity^24–26^. There have also been small-scale demonstrations of superconducting networks and classifiers^26–29^. The work presented here is distinct from these previous efforts because it is self-training and scalable to deeper neural networks. In addition, because of the nature of the self-training approach this architecture has the potential to be used for continual learning.

We take advantage of the SFQ logic family to create simple circuits that can implement reinforcement learning rules and have modified the typical learning rules to follow the constraints of what can be efficiently made with the hardware. In order to complete a superconducting neuromorphic architecture, we have developed a new synaptic function circuit, which controls the strength of the connection between two neurons. We demonstrate that modulating the amplitude of neuronal SFQ pulses can be used to implement the synaptic weighting. Further the weight value of our novel synaptic circuit can be programmed via SFQ spikes that are the output of reinforcement learning logic circuits. With these building blocks, we create a biologically inspired self-training architecture, demonstrate its basic functionality, and explore its ability to be scaled to larger tasks.

While algorithmic neural networks have proven to be useful for a multitude of tasks they are typically limited by the energy and time that it takes to train them^30^. Reinforcement learning has been shown to be a viable method for training neural networks^31^. In its most basic form, reinforcement learning establishes a reward function based on the comparison between the desired and actual outputs of a system^32,33^. We have modified the typical reinforcement learning algorithm to be compatible with our hardware constraints to create this self-training superconducting architecture. We test this architectural approach using accurate SPICE models that have been previously validated with fabricated neuromorphic circuit elements^34^. We demonstrate the basic functionality of the neural network by having it learn several small problems using these SPICE models, demonstrating robust learning capability. We extend these results by implementing in Python the hardware-constrained learning rules in a neural network that learns the MNIST handwritten digit benchmark. The most striking result from this architecture is the training speed of order 1 ns per learning cycle, which we show has favorable scaling to large networks.

## Results

**Fig. 1:**
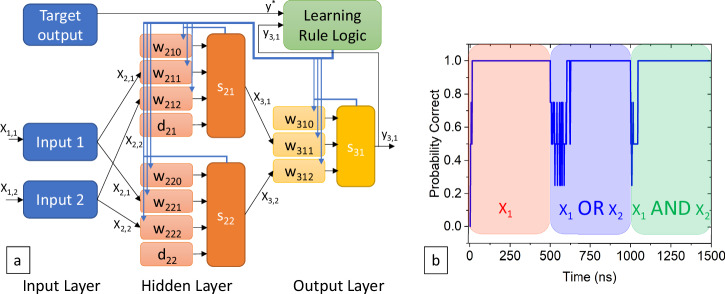
Block diagram of 2-2-1 network, and the network learning three different functions. **a** Block diagram of small scale demonstration network. Further details of the notation and indicies are given in the methods section. **b** Probability correct versus time for a continuous SPICE simulation of the network learning three different functions. The functions are spike when input 1 is 1 for the first 500 ns, spike when either input 1 or input 2 spikes for the next 500 ns, and spike only when input 1 and input 2 spike for the last 500 ns.

We first implemented a SPICE model of a small but complete spiking, reinforcement learning-based self-training superconducting network. This network requires bipolar synaptic circuits, threshold soma circuits, and learning rule logic circuits, the details of which are described in the Methods section. Figure 1a shows a basic block diagram of this network. There are two inputs that each take an external binary signal and convert it to a single flux quantum (SFQ) spike for a 1 and no spike for a 0. In addition, there is an input clock (not shown in the diagram) that spikes on every input cycle and is used for both a bias weight *w*_*0*_ to adjust the threshold and the stochastic excitations (*d)* that are summed along with the weighted inputs in the soma.

The test network modeled in SPICE has two input units fully connected to a hidden layer of two units, which is subsequently fully connected to an output layer with one unit. The block diagram of the network can be seen in Fig. 1a. The details of the weight circuits (*w*), the stochastic excitation circuits (*d*), and summation circuits (*s*) are described in the methods section. Briefly, the weights are bipolar synaptic circuits labeled as *w*_*l,u,i*_ where *l* is the layer, *u* is the unit, and *i* is the input, with *i* = 0 being the bias of a given unit. The summation circuit adds all incoming weighted signals with the bias and stochastic excitation. If the fixed threshold is exceeded the unit spikes and the signal is propagated to the next layer in the network.

The common Error Backpropagation learning rules that are used to adjust the weights in neural networks are based on stochastic gradient descent to minimize the mean squared error between the network’s output and its desired outputs. In this work, we instead implement a correlative update algorithm involving a global reinforcement signal simultaneously sent to all hidden layer units. The algorithm, described in detail in the methods section, is based on Williams’^35^ definition of his non-episodic REINFORCE algorithm with consideration given to the efficient implementation in superconducting circuits. While this type of algorithm generally requires more updates than Error Backpropagation, it considerably reduces the complexity of the circuits required for training. It thus provides advantages in both size and speed in a direct hardware implementation.

Figure 1b shows the results from a single SPICE simulation of the network. The network is trained to classify an input vector by providing the desired output y* for a series of input vectors. In this simulation input 1 had an input series of 0011 repeated and input 2 had an input series of 0101 repeated. The time for inference plus learning in this example was 1 ns. The target output was divided into three phases. In phase 1, which covers the first 500 inputs (500 ns in time), the network learned to spike whenever input 1 spikes (X_1_) regardless of input 2. In the second phase (from input 501 ns to 1000 ns) the network learned to spike when either input 1 or input 2 spiked (OR). In the third phase, from 1001 ns to 1500 ns, the network learned to spike only when input 1 and input 2 spiked (AND). It should be noted that this was a continuous simulation where the only change between the phases was the target output y^*^. This helps illustrate the robustness of the training algorithm to any effect of the initial weight state. The effective training of these three phases can be seen in the graph of the probability correct of Fig. 1b. The probability correct is a running average of four cycles of the probability that the output was correct for a given input vector, meaning that a value of 1 implies that the output of the network matched the target output for at least four cycles. Figure 1b shows that given a new desired output, the network can adjust its own weights without intervention to learn this function. This ability for the network to retrain is quite promising for continual learning applications.

**Fig. 2:**
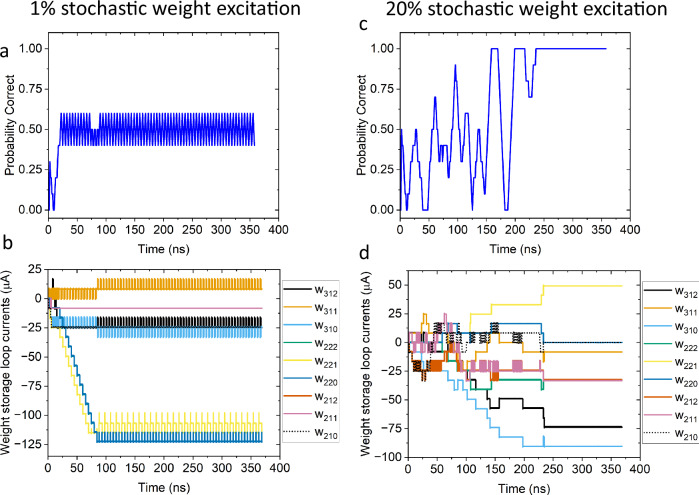
Effect of stochastic weight exploration on learning. **a** probability correct vs. time for the network attemting to learn the X2 function with a problematic input sequence and 1% stochastic excitation magnitude. **b** the weight storage currents (offset for clarity) of the synapses for the simulation in (**a**). **c** probability correct vs. time for the same network and input sequence as in (**a**) and (**b**), but with a stochastic exication magnitude of 20% of the maximum synapse value. **d** the weight storage currents (offset for clarity) of the synapses for the simulation in (**c**).

The previous functions could be robustly learned without stochasticity. However, most non-trivial classifications benefit from the enhanced weight exploration that the additional stochastic term in the hidden layer units provides. Figure 2 shows the same network training to spike whenever input 2 spikes (X_2_). In this example, input 2 was fed an alternating signal of 010101… Without providing the same input for two samples in a row the change in the reward signal *Δr* and the change in the output *Δy* no longer provide a well-defined reinforcement signal for the weight updates to follow. Figure 2a, b have a stochastic excitation of the hidden layer units that was set to <1% of the maximum value of a synaptic weight. Figure 2a shows the probability that the network outputs the correct value. Figure 2b shows the stored weights as current in the storage loop in the synapse. The subscripts of the weights correspond to the numbering scheme described in Fig. 1. It can be seen in this case that the periodic oscillation of the value of input 2 leads to an oscillation of the weight values around a local minimum in the error, which prevents the network from converging on the correct output behavior.

A stochastic excitation can enable training around this flawed input sample, even in this case where there is not a clear reinforcement signal for the hidden units to follow. In our SPICE simulations we have a stochastic input to each soma that is a bipolar synapse as described in the methods section. This stochastic input is tuned to have only three states available -1, 0, 1. The values are randomly selected in the simulation and could be physically implemented using a thermally excitable JJ. For the simulations the synapse gets an input at every cycle, similar to the bias input. The results of adding a stochastic excitation to the soma circuit can be seen in Fig. 2c, d which have the same input pattern and network as Fig. 2a, b. In the simulations for Fig. 2c, d the stochastic excitation synapse has an increased coupling to the soma with a magnitude of approximately 20% of the maximum weighted input value of the standard inputs. As can be seen in Fig. 2c the network now correctly learns to identify input 2 regardless of the input 1 value. In general, we find that a stochastic excitation of between 20% and 50% of the maximum weighted input is an effective magnitude for weight space exploration. It should be noted that if the inputs were sampled twice, *e.g*. 00110011…, X2 could be correctly learned without the additional stochastic excitation, as expected. Also, we note for completeness that all cases in Fig. 1 can be learned with a stochastic excitation of 20% to 50% of the maximum weighted input. In general, as the problems become more complex the additional weight exploration provided by the stochastic excitation to the hidden units may become more beneficial for faster learning convergence^36^.

**Fig. 3:**
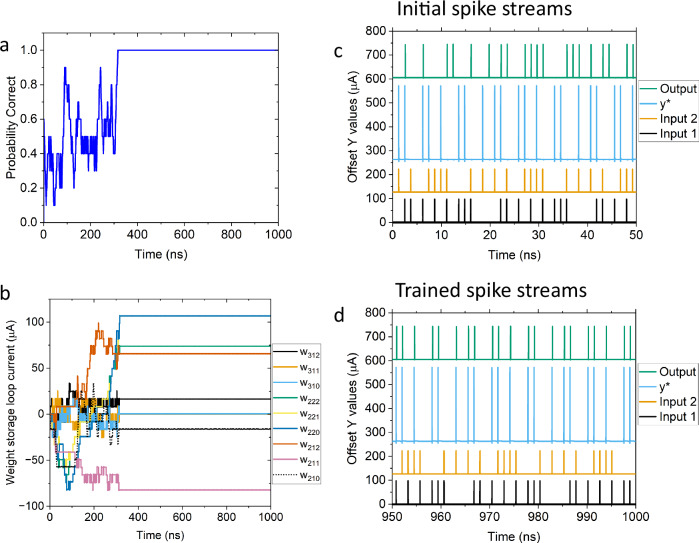
Training results for XOR function. **a** the probability correct versus time for a small network learning the XOR function. **b** the weight storage currents (offset for clarity) of the synapses in this network vs. time. **c** spiking patterns for the input and output of the network at the start of training. **d** spiking patterns for the input and output of the network after the network has learning the XOR funciton showing that the output (y) and target (y^*^) agree.

Figure 3 shows the same network as in Figs. 1 and 2 with a stochastic excitation of about 20% of the maximum weighted input learning the XOR function. This is a nonlinearly separable problem, which therefore requires the hidden layer and demonstrates the generality of both the network and its training rules. In this example we use an input pattern that is a set of 16 quasi-random input vectors that is repeated for 1000 total cycles. Figure 3a shows the probability that the output correctly spikes when only input 1 or input 2 spikes, averaged over 4 cycles. Figure 3b shows the weights of the synapses as the network correctly learns the XOR function in less than 400 ns. Weight subscripts are the same as defined in Fig. 1. Figure 3c shows the spiking pattern of the inputs, the desired output y*, and the actual network output for the first 50 ns of training when the probability correct is approximately 25%. Figure 3d shows the spiking patterns from 950 ns to 1000 ns of the SPICE simulation where one can see that the network has correctly learned the XOR function.

### Extension to MNIST

To investigate the suitability of the reinforcement learning approach for larger problems, simulations were run with a python model implementing the learning rules described in the methods section and applied to the problem of classifying images of handwritten digits in the modified national institute of standards and technology (MNIST) dataset^37,38^. We tried to put limits in the simulations to reflect the constraints of the SPICE models above, these include a weight bit depth of 6 bits and a fan-in limitation of 50. However, details such as cross talk and wiring layout were not considered^38^. The results in Table 1 were obtained with neural networks containing one, two, or three hidden layers, each with 200 units. The networks were trained using 50,000 images for training, 10,000 images for validation and 10,000 images for test as is typical. The training, validation, and testing procedure was performed five times with different random initial weights each time and also a different set of random connections, that arise from the fan-in limitation of 50 detailed below, each time. Table 1 shows the minimum and maximum values of accuracy for the training, validation and test datasets.

**Table 1:** The percentage of train and test set images for MNIST that are correctly classified by networks having no, 1, 2, or 3 hidden layers of 200 units each

Number of Hidden Layers	Minimum Training Set Accuracy	Maximum Training Set Accuracy	Minimum Validation Set Accuracy	Maximum Validation Set Accuracy	Minimum Test Set Accuracy	Maximum Test Set Accuracy
1	80.3%	81.0%	81.3%	82.8%	80.4%	81.9%
2	89.7%	90.7%	90.0%	90.6%	89.3%	90.1%
3	91.1%	92.7%	90.8%	91.3%	90.2%	92.2%

The current hardware design of the soma has a limit of 50 incoming connections^39^. This was modeled in the python implementation in the following way. Each unit in the first hidden layer was connected to a randomly chosen 7 × 7 patch of the 28 × 28 original image. While this is like the kernels in convolutional neural networks, here convolution is not performed. Each unit only gets the 49 intensities from the single 7 × 7 patch randomly assigned to it when the neural network is defined. The units in the remaining hidden layers receive inputs from 49 randomly selected outputs from the previous hidden layer. Each of the 10 units in the output layer receives 49 randomly selected outputs from the last hidden layer. Thus, each unit in the neural network has 49 weights corresponding to its 49 inputs, plus one bias weight, for a total of 50 weights in each unit.

Classification is performed by picking the unit with the largest value among the 10 units in the output layer. Several possibilities exist for implementing this “argmax” operation in the circuit, including a winner-take-all operation based on recurrent connections in the output layer. SPICE modeling of this part of the circuit is beyond the scope of this paper and will be investigated in future work.

### Scaling

In the above examples we show SPICE simulations of a small proof-of-principle self-training network that fully implements reinforcement learning using a common global reward signal. The network is a mixed digital and analog set of circuits that is fully self-contained. The bipolar synapse and soma constitute the analog portion of the network. The analog weights are adjusted via injection of SFQ pulses into a superconducting weight storage loop. SFQ pulses can be injected on either side of the loop, resulting in the ability to increase or decrease the strength of the weight in either direction. The bipolar synapse architecture further enables any synapse to be either inhibitory or excitatory in nature. This bipolar synaptic network style enables a more efficient hardware implementation since synapses do not need to be duplicated or assigned a sign in advance of the training. Because the learning rules determine the direction of a given weight update, there is no need to precisely set the weight value or threshold in the analog portions of the network. This alleviates one of the main challenges in the use of analog hardware, since the typical process variations inherent in all hardware can be accommodated by the learning rules.

Table 2 shows the timing delays based on our SPICE simulations. These timings could likely be reduced with further circuit optimization. In addition, it is worth noting that the 150 ps delay in inference per layer is adjustable based on an L/R time constant in the soma circuit. We choose a 50 ps time constant, which is the approximate time that all incoming spikes to a given soma should arrive. We choose this number to easily account of any jitter or wiring layout non-idealities. Further, this architecture is globally asynchronous. This alleviates many of the issues of clocking SFQ circuits when scaled up.

**Table 2:** Timing delays based on SPICE simulations.

Inference	
Input layer delay	10 ps
Soma delay time per hidden layer	150 ps
Soma delay time output layer	150 ps
Fanout delay time per layer (for layer width L)	6 ps ×ceil (log_2_ *L*)
**Learning**	
Learning update logic delay	750 ps
Fanout delay (for total number of neurons M)	6 ps ×ceil (log_2_ *M*)
Fanout delay (for total number of synapses per neuron N)	6 ps ×ceil (log_2_ *N*)
**Wiring**	
Delay per mm of wire length	10 ps

The total 750 ps learning delay listed in Table 2 comes from three logic updates that each take 250 ps in their current form. These logic cells are the output reward update, the local soma update, and the local synapse update. Since the update rules do not need to be applied sequentially between layers or neurons, the reward signal can be broadcast back to all somas at the same time and the soma rule can be broadcast to all incoming synapses at the same time.

The real advantage of the local learning rules and architecture can be seen when examining how the time delays scale up. We present the 3-layer MNIST network timing as a detailed example. Inference latency is 10 ps input + 48 ps fanout to hidden layer_1_ + 150 ps somas_1_ + 48 ps fanout to hidden layer_2_ + 150 ps somas_2_ + 48 ps fanout to hidden layer_3_ + 150 ps somas_3_ + 23 ps fanout to output layer 100 ps wiring. This gives us 727 ps for the inference latency. The learning update delay is still 750 ps because all updates are independent + 60 ps fanout to all neurons delay + 36 ps fanout to the incoming synapses delay + 100 ps wiring. This gives us 946 ps learning update delay. This gives us a total learning time of 1673 ps per image. Given the 60,000 training images shown twice each for 500 epochs we would expect a total hardware training time of 100.4 ms.

From these two examples we can see three main reasons for the favorable time scaling of the proposed architecture. First, superconducting wiring transmits SFQ signals extremely fast and is not subject to RC time constants. Second, the fanout scales as log_2_ meaning that even a fanout to 1,000,000 units would only add a time delay of 120 ps. Finally, this architecture takes advantage of true local learning rules meaning that the weight updates are independent and globally asynchronous.

**Fig. 4:**
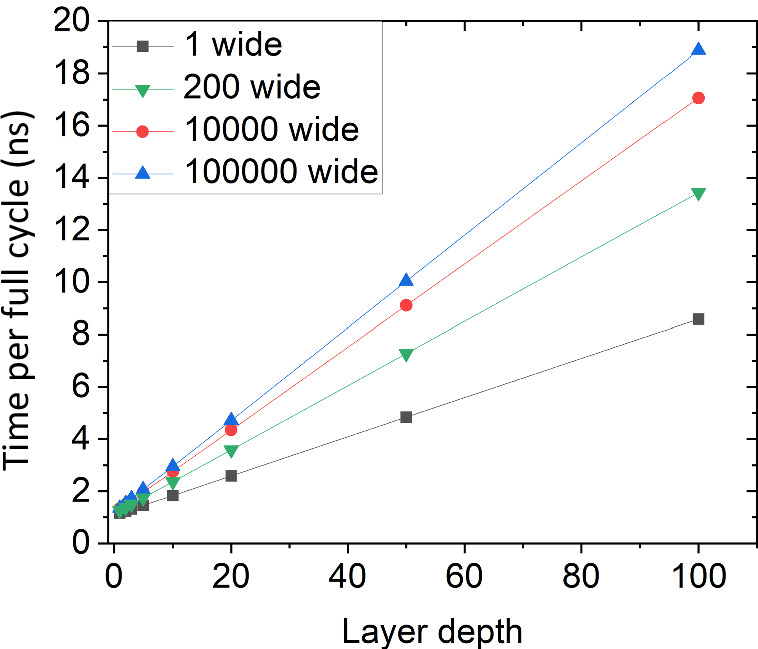
Time scaling of proposed superconducting neuromorphic architecture. Is a graph of the total time delay for inference and learning plus weight update vs. the number of layers in a network for different layer depths.

Figure 4 shows the total time delay for a full inference plus learning cycle versus the number of layers in the network for four different network widths. There is a linear scaling of the inference latency on the number of layers, which dominates the total time as the network grows in size. However, the total time for a learning cycle is still relatively short because of the ability to broadcast the reward signal to the entire network at the same time. While the wiring for this type of network will be a challenge, it does not incur the usual RC time constant or energy issues of typical resistive wiring. It is worth noting that there will be additional timing delays as the network grows due to wiring, but these have a minimal impact on the time scaling. The time to propagate SFQ pulses in Nb is roughly c/3 (where c is the speed of light in vacuum), or 10 ps/mm^40^. While repeater JJs will be needed for JTLs, passive superconducting transmission lines can be used for longer wires. Thus, the additional timing overhead of routing the broadcast signal should be much less than 1 ns. This gives us a 1 million unit, 100 layer deep learning time of about 31 ns or about 30 million cycles per second and favorable time scaling as the network gets larger.

Finally, it is worth noting that million unit networks are not within reach of current fabrication densities. However, our unoptimized example network contained a little less than 1000 JJs for 5 units in total. Using 200 JJs per unit as a very rough estimation, networks with order 1000 units should be achievable with current fabrication technology. This includes the 3 hidden layer MNIST network in our python simulations. If implemented in our JJ reinforcement learning architecture, the 3 hidden layer MNIST network would take about 100 ms as described above. However, after training, image classification could be run at a rate of 1 image every 150 ps (6.7 GHz).

## Discussion

Superconducting neuromorphic computing is relatively undeveloped compared to other emerging devices^5,41^. There are many potential reasons for this. However, the most obvious limitation is the requirement of cryogenic operation, and this should be considered. In this paper we assume a Nb technology that is operational at 4 K. Closed cycle cryogenics has come a long way in recent years expanding potential applications^42,43^. However, edge computing, which many other emerging devices are targeting, is not a good fit for cryogenic operation.

In this paper we have demonstrated the basic components needed to make a complete self-training superconducting neuromorphic network. This includes the analog and digital circuits that are required for basic neural network inference. The digital circuits that are required for learning, and the learning rules that can be efficiently implemented with this hardware. Together these components comprise a superconducting neuromorphic architecture, which we have shown has the potential to scale to more interesting applications. Scaling this architecture past the MNIST level will likely require some further innovations. Of particular interest is finding the best way to work with the fan-in limitation of 50. There are likely architectural choices that mimic the dendritic processing of the brain that can be used to work with this limitation. Future work to increase the capability of these systems has compelling potential.

Even with these limitations, we find that there are many potential advantages that make superconducting neuromorphic computing worth pursuing. High-speed, energy efficiency (if scaled), and natural spiking behavior make superconducting neuromorphic computing a compelling technology to investigate. In this article we have shown a mixed analog and digital neuromorphic SFQ architecture that is self-training using reinforcement learning. The learning rules implemented were constrained for efficient implementation in superconducting hardware. We use SPICE simulations to demonstrate a small-scale network capable of classifying a non-linearly separable problem. We have also demonstrated robust learning with this architecture in part due to the use of stochastic weight exploration in the hidden layer units. We have extended the same reinforcement learning logic demonstrated in the SPICE simulations to python where the network can be scaled to perform MNIST level classifications.

The highlight of this architecture is the ability for the network to self-train given the target output value. This approach mitigates the non-idealities in device variations that is problematic in analog neuromorphic hardware. In addition, the fast-learning time leads to a cycle time of about 1 ns for small networks and excellent time-scaling for larger networks. For example, projections for the total time to train a 3 hidden-layer network to learn the MNIST benchmark are on the order of 100 ms. In addition, after the learning phase, the network can be run in inference only mode, where the same network can operate at one image every 150 ps. These demonstrations prove out the basic building blocks and architecture for a scalable, high-speed network that has inherent fault tolerance. In addition to the speed, the spiking energies of this network are sub-attojoule providing energy efficiency even when accounting for the cooling overhead. Together this architecture provides a clear path and a compelling argument for superconducting neuromorphic computing.

## Methods

### Learning rules

All experiments here are supervised-learning problems for which the desired outputs are known. The common approach for training neural networks to solve supervised learning problems is to use Error Backpropagation to back-propagate the gradient of the mean squared error with respect to every weight. To avoid the extra time and circuits required to perform this backwards flow of information through the multiple hidden layers of a neural network, we instead implement a correlative update algorithm involving a global reinforcement signal simultaneously sent to all hidden layer units. While this requires more updates than Error Backpropagation, it considerably reduces the complexity of the circuits required for training.

The basic definitions and notation used for the network are:

- a network with *L* layers, each layer *l* having *N*_*l*_ units
- weights, *w*_*l,u,i*_ for layer *l*, unit *u*, and input *i*
- bias weights, *w*_*l,u,0*_ for layer *l*, unit *u*
- inputs, *x*_*l,i*_ for layer *l*, and input *i*
- weighted sums, *s*_*l,u*_ for layer *l*, unit *u*
- output *y*_*l,u*_ for layer *l*, unit *u*, which becomes *x*_*l+1,u*_, the inputs to the following layer
- stochastic perturbation *d*_*l,u*_ for layer *l*, unit *u, where d*_*l,u*_ = {*-δ,0,δ*}
- global reward *r*
- weight update *w*_*l,u,i*_ +, with increment size *ρ*
- an input sample consisting of *N*_*1*_ inputs to the first layer, layer 1, with the corresponding desired output y^*^_L,u_, for unit *u* in the last layer, layer L

The equations of the network are slightly different between the hidden layers and the output layer. The output layer is where the global binary reinforcement signal is determined and the target of the output of the units is known directly. The output layer uses its current inputs *x*_*L,i*_, outputs *y*_*L,u*_ and desired outputs y^*^_L,u_ to update its weights. The hidden layers need the current inputs and outputs, a global reinforcement signal, and a memory of the previous output state in order to determine the weight update direction. They also require a stochastic excitation or inhibition input to ensure that the network effectively samples the available states. We find that best practice for learning with this network is for a sample (inputs and desired output) to remain fixed for two time increments before changing conditions. We define the equations of the network as follows:

**Equations for the output layer units**

1 $${s}_{L,u}={w}_{L,u,0}+\mathop{\sum }\limits_{i=1}^{{N}_{L-1}}{x}_{L,i}{w}_{L,u,i}$$

2 $${y}_{L,u}=\left\{\begin{array}{l}1,\;{if}{s}_{L,u}\,>\,0\\ 0,\;{otherwise}\end{array}\right.$$

3 $${w}_{L,u,i}+=\rho \left({y}_{L,u}^{* }-{y}_{L,u}\right)\left({x}_{L,i}-0.5\right),{\rm{for}}i\,>\,0$$

4 $${w}_{L,u,0}+=\rho \left({y}_{L,u}^{* }-{y}_{L,u}\right)$$

5 $$r=\left\{\begin{array}{c}1,{if\; network}^{\prime}s\,{outputs\; are\; all\; correct}\\ 0,{otherwise}\end{array}\right.$$

6 $$\Delta r={r}^{{now}}-{r}^{{previous}}$$

**Equations for hidden layer units**

7 $${x}_{l,i}={y}_{l-1,i}{for\,l}=1,2,3,...$$

8 $${s}_{l,u}={w}_{l,u,0}+\mathop{\sum }\limits_{i=1}^{{N}_{l-1}}{x}_{l,i}{w}_{l,u,i}+{d}_{l,u}$$

9 $${y}_{l,u}=\left\{\begin{array}{l}1,\;{if}{s}_{l,u}\,>\,0\\ 0,\;{otherwise}\end{array}\right.$$

10 $$\Delta {y}_{l,u}={y}_{l,u}^{{now}}-{y}_{l,u}^{{previous}}$$

11 $${w}_{l,u,i}+=\rho \Delta r\Delta {y}_{l,u}\left({x}_{l,i}-0.5\right),{\rm{for}}\,i\,>\,0$$

12 $${w}_{l,u,0}+=\rho \Delta r\Delta {y}_{l,u}$$

The above equations for updating the hidden layer units are based on Williams’^35^ definition of his non-episodic REINFORCE algorithm, given by:

13 $$\Delta w=\alpha \left(r-\bar{r}\right)\left(y-\bar{y}\right)$$

14 $${\bar{r}}_{t}=\gamma {r}_{t-1}+\left(1-\gamma \right){\bar{r}}_{t-1}$$

15 $${\bar{y}}_{t}=\gamma {y}_{t-1}+\left(1-\gamma \right){\bar{y}}_{t-1}$$

Williams describes these equations as pertaining to a bias weight having a constant input of 1. With the addition of variable inputs, ${x}_{j}$, and using $\gamma =1$, these equations reduce to

16 $$\Delta{w}_{j}=\alpha \left(r-{r}_{t-1}\right)\left(y-{y}_{t-1}\right)\left({x}_{j}-1/2\right),$$

which correspond to the weight update equations given above for the hidden layer units. Letting *W* represent all of the weights in the network, Williams proves for his REINFORCE class of algorithms, that the inner product of $E\left\{\Delta \left.W\right|W\right\}$ and $\nabla E\left\{\left.r\right|W\right\}$ is nonnegative. While this is not a convergence proof, it does prove that the change in weight values, $\Delta w$, tends to increase the expected value of *r*.

A known limitation of Williams’s REINFORCE algorithm is that it learns more slowly than a version that includes a learned value function^44^. In future work, a learned value function could be added using a second neural network implemented and trained similarly to our current neural network implementation.

### Single flux quantum circuit implementation of the learning rules

The weight update rules for the output layer can be implemented with standard rapid single flux quantum (RSFQ) digital logic gates^6,45^ and a learning clock signal whose only requirement is to arrive any time after the inference output. While there are many potential ways to implement the logic, we used the following basic approach. Weight update values that are potentially zero, for example (y^*^-y), are used as the clock signal for the logic gates that are closer to the synapse. The absence of this clock ensures that there is no unwanted weight update. The sign of any prefactor is determined as close as possible to the output layer. For example, $\Delta r\Delta y$ is determined at the soma output where ${y}^{{now}}$ is stored during inference. The final sign of the update is determined at the synapse since it depends on the input *x* to the synapse. Finally, all values that are stored from the inference step are reset at the end of the learning cycle to prepare for the next inference cycle. Approaching the update logic in this way concentrates the number of logic gates at the output and soma level, minimizing the number of gates at the synapse and the amount of information that must be broadcast to each synapse.

**Fig. 5:**
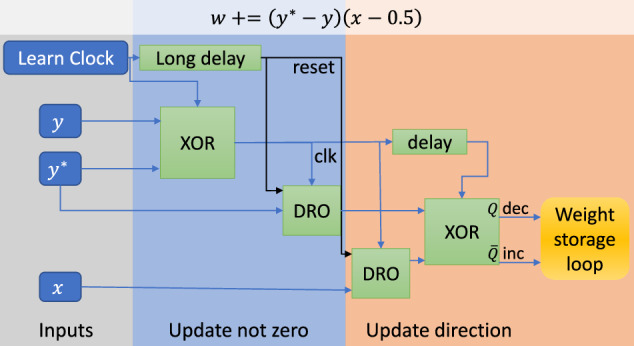
Block diagram of weight update logic. Is a block diagram of weight update logic for a synaptic weight in the output layer.

Figure 5 shows an example of the nominal weight update logic for an output layer synapse. The desired output y* and network output *y* are fed into an exclusive or (XOR) gate to determine if there is a weight update. If the values are the same, (y^*^- y) = 0 there should be no update. Otherwise, the signal from this XOR is used as a clock for the rest of the update logic. To determine the sign of the weight update, the input to the synapse *x* and the desired output y^*^ are fed into an XOR gate via destructive readout (DRO) gates to determine the direction of the update. The output and complementary output of this XOR feed a single flux quantum (SFQ) pulse into the decrement or increment side respectively of the weight storage loop described below in the synapse section. A similar logical flow is followed for the other weight updates.

**Fig. 6:**
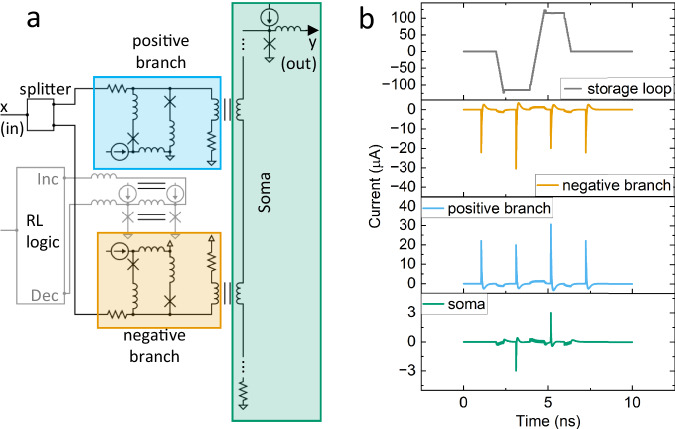
Bipolar synaptic circuit and demonstration of weights affect the spiking magnitude in the soma circuit. **a** bipolar synapse conceptual circuit diagram. Synapse portion in red box for positive weight, synapse portion in green box for negative weight. Blue wiring for the weight adjustment controlled by the reinforcement learning (RL) circuits described below. The two parts of the synapse are both coupled into the soma circuit, which can couple together multiple synapses. **b** SPICE simulation results showing the response of the synapse branches and soma to input spikes that occur when the storage weight is at zero, minimum, maximum, and after returning to zero.

**Bipolar synapse and soma circuit** While the neuron and axon building blocks are easily implemented with simple superconducting elements, the required weight and threshold functions are implemented using an analog circuit that we break up into the synapse (weighting operation) and soma (summation operation)^5,11^. The synaptic subcircuit in Fig. 6a is composed of a bipolar “synaptic” weight, meaning that the weight can be either positive or negative, connected to a summation “soma.” The soma contains a line of mutually coupled inductors with a resistor to ground at one end and the input to a Josephson transmission line at the other. The inputs are set to arrive at the same time where they are weighted, and a portion of the incoming spikes are coupled into the soma. The resistor to ground at the bottom of the soma can be used to adjust the L/R time constant of this summation operation to account for wiring and jitter on the incoming signals, relaxing the timing condition on spikes arriving to the soma. The top of the soma feeds a biased JJ, which then applies the threshold activation function defined above as *y*_*L,u*_. One technical difference is that in the circuit implementation, the threshold value is shifted from zero current in the soma to a positive current value. The threshold is set by a combination of the fixed bias current of the JJ at the top of the soma that is the input to the Josephson transmission line, and the *w*_*0*_ bias weight, which is adjusted by the reinforcement learning logic. The effect however is the same; if the threshold is exceeded a single flux quantum (SFQ) pulse is emitted by the soma (1), otherwise nothing is emitted (0).

A more detailed look at the synapse shown in Fig. 6a follows: The weighting is accomplished using an inductive divider, where one path passes through an adjustable superconducting quantum interference device to ground, and the other path passes through an inductor in series with a resistor to ground. This second path’s inductor is mutually coupled to the soma along with the other synapses that are connected to that soma. The resistor on the second leg of the divider is used to prevent unintended current buildup and to set the L/R time constant (leak rate) of the input signal to a synapse. The weight storage loop (shown in grey wiring), controlled by the learning logic, affects the inductive divider by changing the amount of current circulating in adjustable SQUID, which in turn changes its inductance. Each synapse has a positive and negative branch. Both branches have the same inductive divider topology described above but with the coupling directions changed.

Figure 6b shows the behavior of the synapse from SPICE simulations. The storage loop starts at zero current and an input pulse *x* is applied to the synapse. In this case the positive and negative branches have equal and opposite current coupled into the soma, which is seen as no spike appearing in the soma at the bottom of Fig. 6b. Note that while perfect cancelation is accomplished in the simulation, it is not required to train the network. After the first input spike, a series of 32 SFQ pulses is applied to the decrement side of the weight storage loop in about 1 ns. Note that the number of SFQ pulses stored in the weight storage loop has a maximum of 30 for the simulated circuit shown here with a storage inductor of 250 pH. When the maximum number of SFQ pulses for the weight storage loop is exceeded, the additional SFQ pulses are expelled via the JJ on the other side of the loop and the maximum current value in the weight storage loop is maintained. After this minimum value of the weight storage loop is reached, a subsequent input pulse *x* is applied to the synapse at 3 ns and now a larger portion of the negative branch and a smaller portion of the positive branch are coupled into the soma. This is seen in the soma at the bottom of Fig. 6b as the net negative spike. After this, 64 SFQ pulses are applied to the increment side of the weight storage loop. Subsequently an input pulse *x* is applied to the synapse, which results in a larger positive and smaller negative spike. This is seen in the soma as a net positive spike from the input *x*. Finally, 30 pulses are applied to the decrement side of the weight storage loop to return the weight storage loop to near zero current. The final input pulse *x* is applied to the synapse and once again the positive and negative branches cancel out and no spike is observed in the soma.

One other element that is used is a *w*_*0*_, which acts as an adjustable bias for the soma. The *w*_*0*_ circuit is the same as the positive branch of the bipolar synapse circuit. Each soma circuit has one such *w*_*0*_ to adjust the threshold level. There is a separate weight loop and its own learning rule logic to set and store this *w*_*0*_ for each soma. In addition, there is a *w*_*0*_ input that spikes at every inference cycle. This results in the bias value being independent of whether an input signal was sent to a particular neuron, and no external adjustment of the soma bias is needed as described in the network equations above.

The weight storage is a superconducting loop with a biased JJ on either side, shown in light blue in the middle of Fig. 6a. Current can be injected in either direction in this loop following the result of the learning rules. The size of the inductor sets the effective bit depth of the weight storage. In addition, the adjustable SQUIDs in the synapses are set so that they always remain in the non-spiking state regardless of the weight value, which results in a smooth variation in weight value. In the simulations described in this article we use a 250 pH storage inductor, which results in just under a 6-bit weight. The bit depth is approximately linear with inductor size, meaning that one can use a 500 pH storage inductor for a weight depth of about 6 bits.

## Data Availability

The MNIST dataset if available here: https://git-disl.github.io/GTDLBench/datasets/mnist_datasets/. The datasets used and/or analyzed during the current study are available from the corresponding author on reasonable request.

## Code availability

The underlying code for this study is not publicly available but may be made available to qualified researchers on reasonable request from the corresponding author.
